# The inhibition of protein translation promotes tumor angiogenic switch

**DOI:** 10.1186/s43556-022-00081-4

**Published:** 2022-06-13

**Authors:** Hui Luo, Yuge Shen, Weiting Liao, Qiqi Li, Ni Wu, Jian Zhong, Chaoxin Xiao, Jia Gan, Yun Yang, E. Dong, Guimin Zhang, Binrui Liu, Xiaozhu Yue, Lin Xu, Yan Liu, Chengjian Zhao, Qian Zhong, Hanshuo Yang

**Affiliations:** 1 State Key Laboratory of Biotherapy and Cancer Center, West China Hospital, Sichuan University and Collaborative Innovation Center, No.17 Renmin South Road Section Three, Chengdu, 610041 Sichuan China; 2 The Third Affiliated Hospital of Chengdu Medical College,Pidu District People’s Hospital, Chengdu, China; 3 Department of Gynecology and Obstetrics, West China Second University Hospital of Sichuan University, Chengdu, China; 4 Experimental and Research Animal Institute, Sichuan University, Chengdu, China

**Keywords:** Angiogenic switch, HIF, Microtumor, Protein translation, *Vegfa*

## Abstract

The ‘angiogenic switch’ is critical for tumor progression. However, the pathological details and molecular mechanisms remain incompletely characterized. In this study, we established mammal xenografts in zebrafish to visually investigate the first vessel growth (angiogenic switch) in real-time, by inoculating tumor cells into the perivitelline space of live optically transparent Transgenic (*flk1:EGFP*) zebrafish larvae. Using this model, we found that hypoxia and hypoxia-inducible factor (HIF) signaling were unnecessary for the angiogenic switch, whereas vascular endothelial growth factor A gene (*Vegfa*) played a crucial role. Mechanistically, transcriptome analysis showed that the angiogenic switch was characterized by inhibition of translation, but not hypoxia. Phosphorylation of eukaryotic translation initiation factor 2 alpha (Eif2α) and the expression of *Vegfa* were increased in the angiogenic switch microtumors, and 3D tumor spheroids, and puromycin-treated tumor cells. *Vegfa* overexpression promoted early onset of the angiogenic switch, whereas *Vegfa* knockout prevented the first tumor vessel from sprouting. Pretreatment of tumor cells with puromycin promoted the angiogenic switch in vivo similarly to *Vegfa* overexpression, whereas *Vegfa* knockdown suppressed the increase*.* This study provides direc and dynamic in vivo evidences that inhibition of translation, but not hypoxia or HIF signaling promotes the angiogenic switch in tumor by increasing *Vegfa* transcription.

**Supplementary information:**

The online version contains supplementary material available at 10.1186/s43556-022-00081-4.

## Introduction

The tumor angiogenic switch, whereby normally quiescent vasculature grows new capillaries, separates the avascular phase characterized by a dormant tumor and the vascular phase in which exponential tumor growth ensues [1]. The angiogenic switch is an adaptive response that is believed to overcome the growth limitation from an inadequate supply of nutrients and oxygen [2–4]. The new capillaries converge toward the tumor and contribute to tumor progression not only by providing oxygen and nutrients for tumor outgrowth, but also by offering a route for tumor cells to disseminate to distant organs and form metastases. Moreover, neovasculature plays important role in molding a suppressive immune microenvironment within tumors. The combination of anti-angiogenic and immune checkpoint blockers has achieved promising results in cancer therapy [5–8].

HIF is an oxygen-sensitive transcription factor that is believed to be a master regulator of the angiogenic switch [9], by inducing gene expression of proangiogenic factors such as VEGFA [10], fibroblast growth factor, and platelet-derived growth factor [11, 12]. Accumulated HIF-1 upregulates the gene expression of matrix metalloproteinase, integrins, and some pro-angiogenic receptors that are essential to sprout new vessels [13–16]. VEGFA is considered to be the most important and best-studied proangiogenic factor [17, 18]. VEGFA is widely expressed by tumor cells and acts through VEGF receptors to induce angiogenesis, by increasing microvascular permeability, promoting endothelial cell survival, division and migration, and prevent senescence, etc. [19].

Traditional experimental models of investigating angiogenesis include the corneal micropocket, chick chorioallantoic membrane, rodent mesentery, and subcutaneous sponge/matrix/ matrigel plug/alginate microbead in mice [20, 21]. Each model or technology has its advantages and disadvantages. An ideal experimental system to investigate the angiogenic switch should possess the following characteristics: high resolution at the single cell level, appropriate for real-time observation and quantitative analysis, ability to display the critical process of the transition from the avascular to vascular stage, and ease of establishment and manipulation. However, it is almost impossible for the currently used models to precisely and directly distinguish the avascular and vascular status in tumors during the dynamics of the angiogenic switch. The mechanism that unambiguously induces growth of the first vessel sprouting from the host vascular system into a tumor has not been directly visualized in vivo. Therefore, the involvement of hypoxia, HIF, or VEGF in the angiogenic switch remains inconclusive.

Zebrafish is a promising animal model for tumor angiogenic switch research. The transparency of zebrafish embryos and the availability of vascular-specific transgenic reporter lines with enhanced green fluorescent protein in all blood vessels throughout embryogenesis [22, 23] allow easy intravital imaging of vessels. We have established a xenograft tumor model in zebrafish perivitelline space. This model can be used to dynamically visualize tumor angiogenesis in vivo at high-resolution without surgical or other invasive procedures [24–26]. Here, we developed this model to investigate the angiogenic switch in which the first tumor vessel sprouting from host vasculatures was clearly visualized in vivo. We found that inhibition of protein translation, but not hypoxia or HIF, promoted the angiogenic switch in tumor by increasing *Vegfa* transcription.

## Results

### Angiogenic switch model in zebrafish

**Fig. 1:**
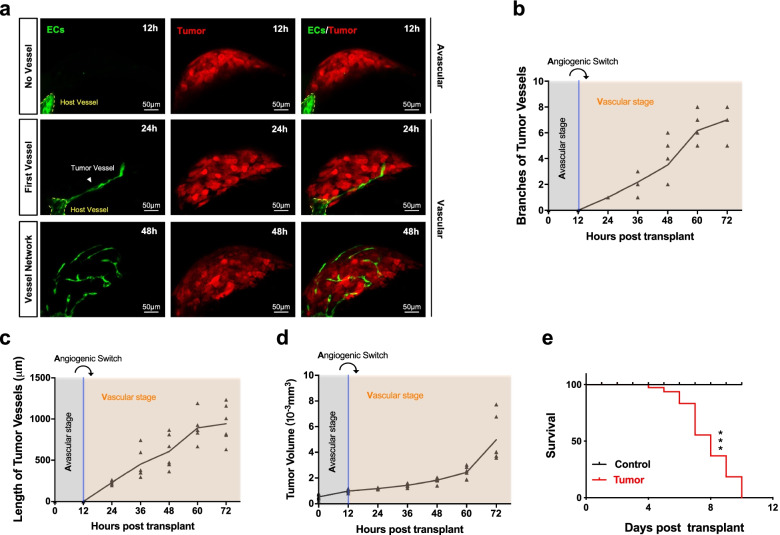
Angiogenic switch model in zebrafish. **a** Imaging of the tumor vascular growth with a confocal microscope. The first column indicates that the transplant microtumor does not yet have blood vessels (No vessel, Avascular phase). The second and third columns indicate the presence of blood vessels in the transplant microtumor (First vessel, Vessel network; Vascular phase). Endothelial cells (ECs) are green. Tumor is red. Yellow line, host vessel. White arrow, tumor vessel. Scale bar, 50 μm. **b** The branches and **c** length of tumor vessels and **d** tumor volume post tumor cells transplant for 72 h (*n* = 6). Light blue shade, avascular stage. Light orange shade, vascular stage. Light black curved arrow, angiogenic switch. **e**. Survival curve for zebrafish with or without transplant tumor cells for 10 days (*n* = 20). Log-rank (Mantel-Cox) test, *** *P* < 0.001

To visually investigate the angiogenic switch, we established an angiogenic switch model by inoculating B16-Red cells into the perivitelline space of live optically transparent Tg(*flk1:EGFP*) zebrafish larvae (Fig. 1a, Supplementary Fig. 1a). Successful tumor growth was confirmed by H&E staining at 7 days post-injection (dpi) (Supplementary Fig. 1b). In this model, tumor cells are labelled with red fluorescence, and endothelial cells are labelled with green fluorescence. Thus, the angiogenic switch was observed under a confocal microscope at high resolution (Fig. 1a). The no vessel phase (approximately 12 h post-injection) represents the avascular state, whereas the first vessel phase (approximately 24 h post-injection) represents the onset of the vascular state, after which the vessel network forms (Fig. 1a). The transition from no vessel to the first tumor vessel phase is the angiogenic switch (Fig. 1a). The number of tumor vessel branches increased (Fig. 1b), the length extended (Fig. 1c), and the volume increased (Fig. 1d). The mortality of zebrafish significantly increased over time post-injection compared with control fish (Fig. 1e). This experimental evidence suggested the successful establishment of the zebrafish angiogenic switch model.

### Angiogenic switch does not depend on hypoxia or HIF-1 signaling

**Fig. 2:**
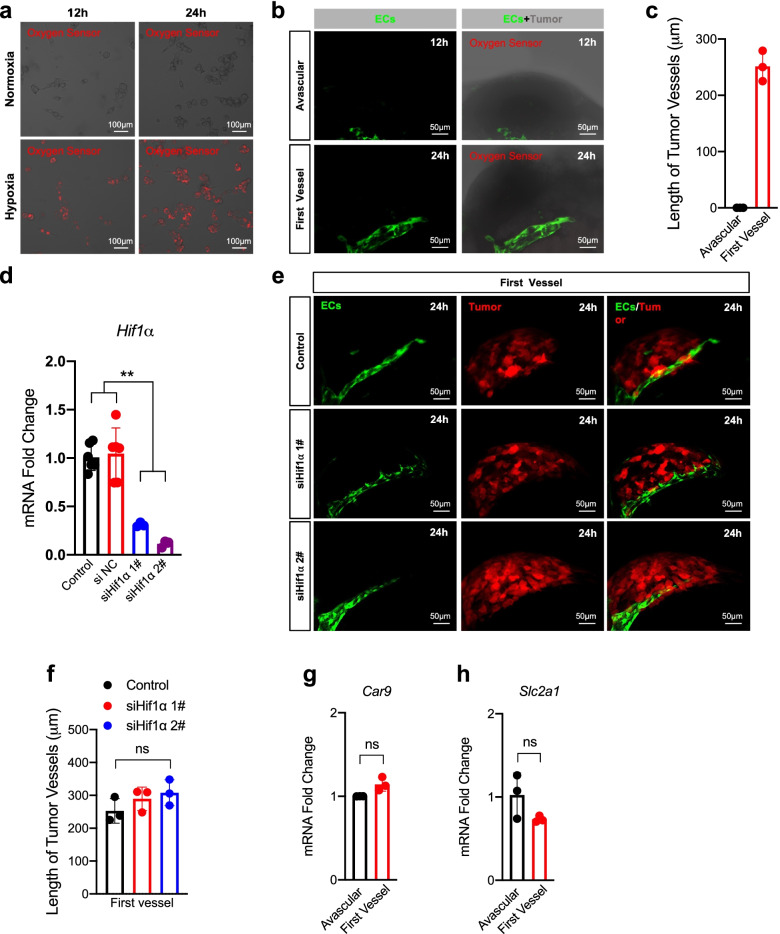
The angiogenic switch does not depend on hypoxia or *Hif-1α*. **a** Imaging of B16 cells incubated with hypoxia probe reagent (oxygen sensor) cultured under hypoxic or normoxia. Scale bar, 100 μm. **b** Imaging of the hypoxia probe reagent incubated transplant B16 microtumors under normoxia. Tumor is grey. Scale bar, 50 μm. **c** The length of the first vessel of the B16 microtumors under normoxia. **d** RT-qPCR quantification of the *Hif1α* expression in B16-Red cells transfected with *Hif1α* siRNA (50 μM) for 48 h (*n* = 3). NC, negative control. **e** Imaging of the first tumor vessel growth of *Hif1α* knockdown B16-Red transplant microtumors. Tumor is red. Scale bar, 50 μm. **f** The length of the first tumor vessel of *Hif1α* knockdown B16-Red transplant microtumors. **g** and **h**. RT-qPCR quantification of the *Car9/CA9* and *Slc2a1/GLUT1* expression in the transplant microtumors during the angiogenic switch (about 12 h and 24 h). Red fluorescence, hypoxia. ECs are green. Unpaired t-test, ns *P* > 0.05, *** *P* < 0.001 (*n* = 3–5)

Hypoxia stimulates the formation of tumor blood vessels [27] and functions as an angiogenic master switch by the activation of HIFs [9]. We examined whether hypoxia had occurred using a red hypoxia reagent during the angiogenic switch. We confirmed that the B16 cells incubated with the hypoxia reagent under hypoxia (1%) showed fluorescence, whereas cells under normoxia did not (Fig. 2a). No fluorescence was found in the hypoxia reagent-treated B16 microtumors (Fig. 2b) and the length extended (Fig. 2c). To verify these results, we knocked down *Hif1α* in B16-Red cells by siRNA (Fig. 2d, Supplementary Fig. 2a). We implanted *Hif1α* knockdown tumor cells in zebrafish and found no significant effect on the angiogenic switch (Fig. 2e). The length (Fig. 2f) showed no significant changes in the *Hif1α* knockdown tumor compared with that in the control. However, *Hif1α* knockdown inhibited the increase in gene expression and protein levels of *Vegfa* under hypoxia (Supplementary Fig. 2b-c). Because *Car9*/*CA9* (HIF-1-specific target gene) and *Slc2a1/GLUT-1* (HIF-2 target gene) are commonly used as downstream markers of HIF signaling and characterize activation of the HIF pathway [28], we detected their expression, which showed significant changes at a late stage of angiogenesis (Supplementary Fig. 3a-b) but no significant changes during the angiogenic switch in microtumors (Fig. 2g-h).

**Fig. 3:**
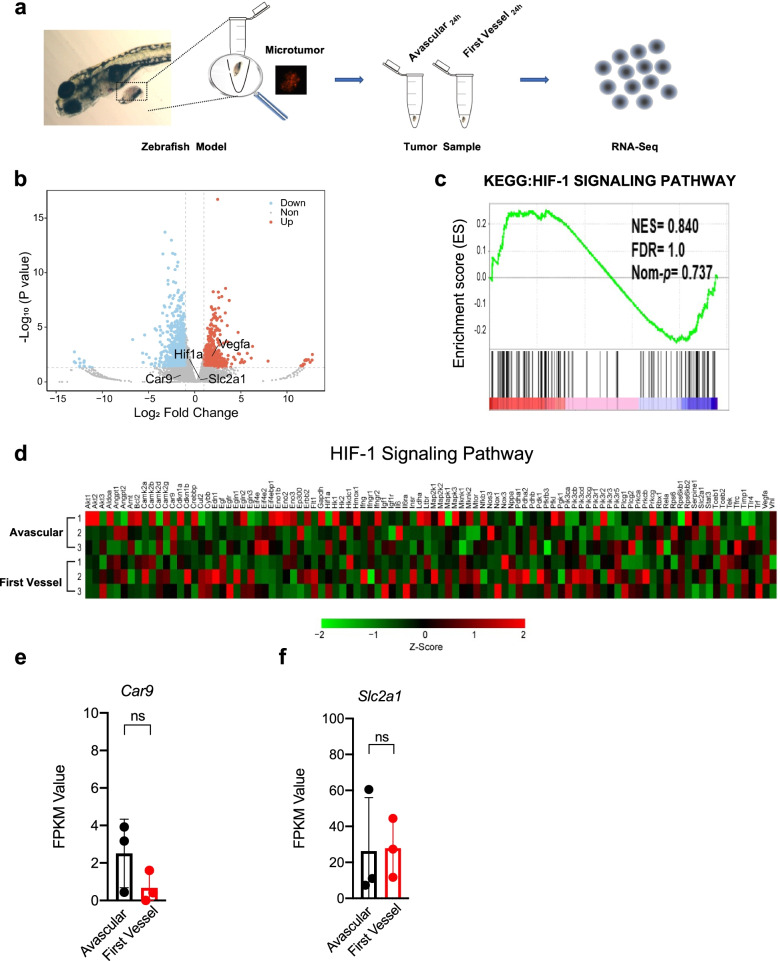
The angiogenic switch does not depend on HIF-1 signaling. **a** Schematics indicating the microsurgery of dissecting the transplant microtumors and transcriptome sequencing. Avascular, the tumor has not yet induced angiogenesis, which means ‘angiogenic switch’ is off (about 12 h); First vessel, the tumor induces the sprouting of the first vessel, which means ‘angiogenic switch’ is on (about 24 h). **b** The volcano plot of the gene expression change of the dissected transplant microtumors transcriptome during the angiogenic switch (*n* = 3). **c** The GSEA analysis and **d** the heat map of the HIF-1 signaling pathway during the angiogenic switch (*n* = 3). **e** and **f** The change of FPKM value of RNAseq data for *Car9/CA9* and *Slc2a1/GLUT1*

Microtumors in the avascular state and the first vessel state (before or after the angiogenic switch) were dissected by microsurgery and sequenced by RNA-seq (three replicates) (Fig. 3a). The volcano plot showed that gene expression was significantly up-regulated or down-regulated (|Log2 Fold change|≥ 1.5) during the angiogenic switch (Fig. 3b). However, GSEA analysis showed that the HIF-1 signaling pathway did not change significantly (NES 0.840, FDR 1.0, and Nominal p-value 0.737) (Fig. 3c), and similar findings were shown by a heat map (Fig. 3d). Moreover, expression of HIF-2-specific target genes *Serpine1/PAI1* and *Epo* showed no significant changes during the angiogenic switch in transplant microtumors (Supplementary Fig. 3c-d). Additionally, there were no significant changes in expression of other HIF target genes during the angiogenic switch (Fig. 3e-f, Supplementary Fig. 4a-k). Thus, the angiogenic switch did not depend on hypoxia or HIF-1 signaling.

### Angiogenic switch depends on *Vegfa*

**Fig. 4:**
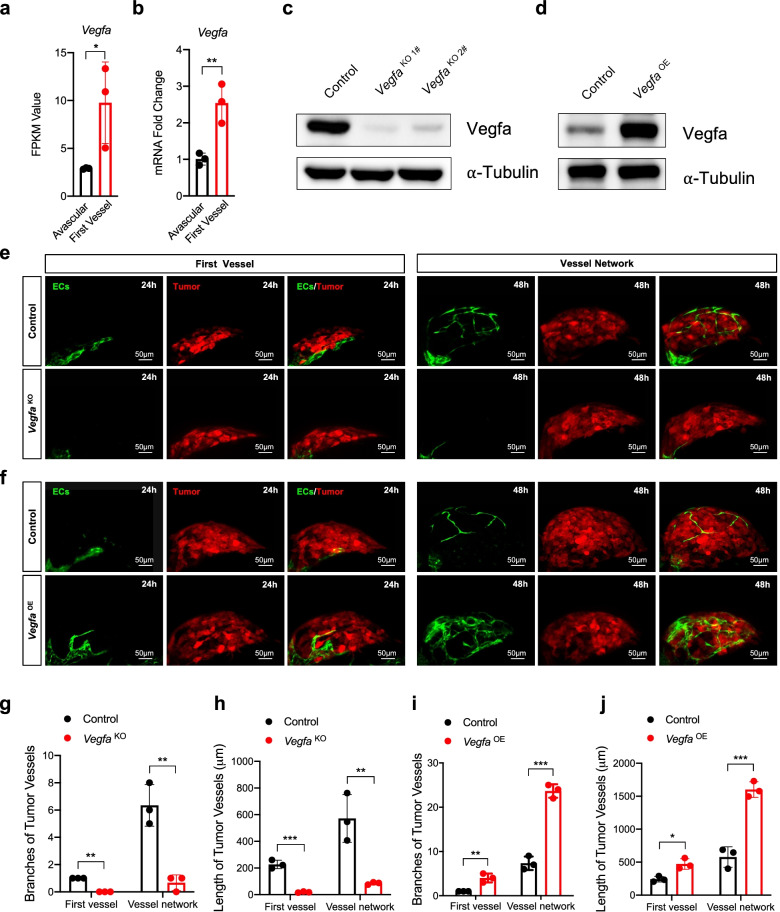
Angiogenic switch depends on *Vegfa*. **a** The change of FPKM value of RNA-seq data for *Vegfa*. **b** RT-qPCR quantification of *Vegfa* expression in the transplant microtumors during the angiogenic switch (about 12 h and 24 h). **c** and **d** Western blot analysis of Vegfa and α-tubulin of B16-Red cells transfected with LentiCRISPRv2-*Vegfa*, sg1# (*Vegfa*^KO 1#^) or sg2# (*Vegfa*^KO 1#^) knockout plasmid or pcDNA3.1-VEGFA overexpression plasmid (*Vegfa*^OE^) for 48 h. **e** and **f** Imaging of the transplant tumor vascular growth of *Vegfa* knockout or overexpression B16-Red microtumors. ECs are green. Tumor is red. Scale bar, 50 μm. **g-j** The branches and length of the first vessel and vessel network. Unpaired t-test, *P < 0.05, ** *P* < 0.01, *** *P* < 0.001 (*n* = 3–5)

VEGFA is the main and best-studied member of the VEGF family [17], which is regulated by HIF1α [10, 29]. We next examined the role of *Vegfa* during the angiogenic switch. We analyzed the expression of *Vegfa* and a significant increase in the transcriptome (Fig. 4a) and dissected transplant microtumors (Fig. 4b) during the angiogenic switch. We then transfected B16-Red cells with a plasmid to knockout or overexpress *Vegfa* (Fig. 4c-d). We established zebrafish angiogenic switch models with these B16-Red cells and found that *Vegfa* knockout in microtumors prevented even the first tumor vessel sprouting (Fig. 4e), wheres overexpression of *Vegfa* promoted early onset of the angiogenic switch (Fig. 4f). Compared with control tumors, tumors in which *Vegfa* was knocked out had fewer vessel branches (Fig. 4g) and shorter vessel lengths (Fig. 4h), whereas the tumors in which the *Vegfa* was overexpressed had more vessel branches (Fig. 4i) and longer vessel lengths (Fig. 4j). Additionally, we analyzed angiogenesis-related genes in the transcriptome (*Vegfb*, *-c*, *Fgf2*, *Hgf*, *Ang1*, *Ang2* and *Tsp1*) and found no significant differences during the angiogenic switch (Supplementary Fig. 5a-g). Next, we collected microtumors in three states (avascular, first vessel and vascular network) and found that expression of *Vegfa* was significantly increased in all three states (Supplementary Fig. 5h), while expression of *Vegfc*, *Fgf2*, *Hgf*, *Ang1* and *Tsp1* had no significant changes during the angiogenic switch, but had significantly increased in the vascular network state (Supplementary Fig. 5i-m). However, expression of *Vegfb* and *Ang2* had decreased for unknown reasons (Supplementary Fig. 5n-o). These results suggest that the angiogenic switch depends on *Vegfa*.

### Inhibition of protein translation occurrs during the angiogenic switch

**Fig. 5:**
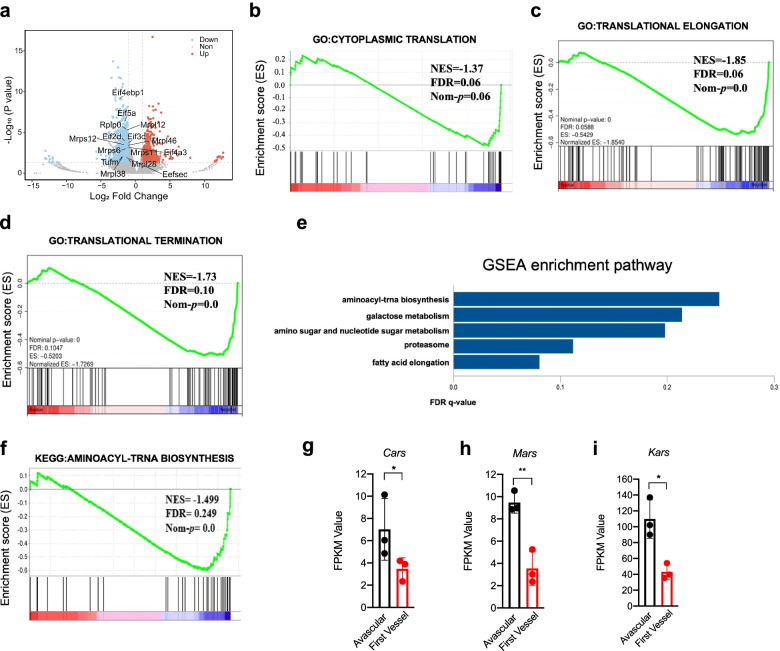
Inhibition of protein translation occurred during the angiogenic switch. **a** The volcano plot of the gene expression change of the dissected transplant microtumors transcriptome during the angiogenic switch (*n* = 3). **b**-**d** The GSEA analysis of transcriptome for the cytoplasmic translation, translational elongation and translational termination gene sets (*n* = 3). **e** The GSEA analysis of transcriptome for the significantly changed pathways during the angiogenic switch (*n* = 3). **f** The GSEA analysis of transcriptome for the aminoacyl-trans biosynthesis pathway during the angiogenic switch (*n* = 3). **g**-**i** The change of FPKM of RNAseq data for some aminoacyl-tRNA synthetases of the aminoacyl-trans biosynthesis during the angiogenic switch. Unpaired t-test, *P < 0.05, ** *P* < 0.01 (*n* = 3)

We found significant downregulation of several genes involved in protein translation (Fig. 5a). Gene Ontology (GO) analysis of the transcriptome during the angiogenic switch indicated that the biological process was mainly related to translation (Supplementary Fig. 6a), the cellular component was mainly related to the ribosome (Supplementary Fig. 6b), and the molecular function was related to the structural constituents of ribosomes (Supplementary Fig. 6c). GSEA revealed that the cytoplasmic translation gene set tended to be down-regulated (Fig. 5b). Additionally, translational elongation and termination gene sets were significantly downregulated (Fig. 5c-d). Moreover, GSEA also revealed five significantly changed KEGG pathways (FDR < 0.25, P-value < 0.05) (Fig. 5e) during the angiogenic switch: aminoacyl-tRNA synthetases, galactose metabolism, amino sugar and nucleotide sugar metabolism, proteasome and fatty acid elongation. Gene expression of the aminoacyl-trans biosynthesis pathway was decreased significantly (Fig. 5f), and similar results were observed for some aminoacyl-tRNA synthetases (Fig. 5g-i). The aminoacyl-tRNA synthetases of the aminoacyl-trans biosynthesis pathway are exquisitely adapted to covalently link a single standard amino acid to its cognate set of tRNA isoacceptors and function in the first step of protein translation [30]. Our results suggested that protein translation in microtumors decreased during the angiogenic switch. Additionally, the gene expression of the galactose metabolism (Supplementary Fig. 6d), amino sugar and nucleotide sugar metabolism (Supplementary Fig. 6e), and proteasome pathways (Supplementary Fig. 6f) was decreased significantly. This would lead to reduced nucleotide sugar production and protein degradation. Nucleotide sugars are precursors to the sugars involved in protein glycosylation [31]. Therefore, protein glycosylation may be impaired during the angiogenic switch. This may be a chain reaction caused by reduced protein translation. Overall, the above experimental evidence suggested that inhibition of protein translation occurred during the angiogenic switch.

### Reduction of protein translation promotes *Vegfa* expression independently of *Hif-1α*

**Fig. 6:**
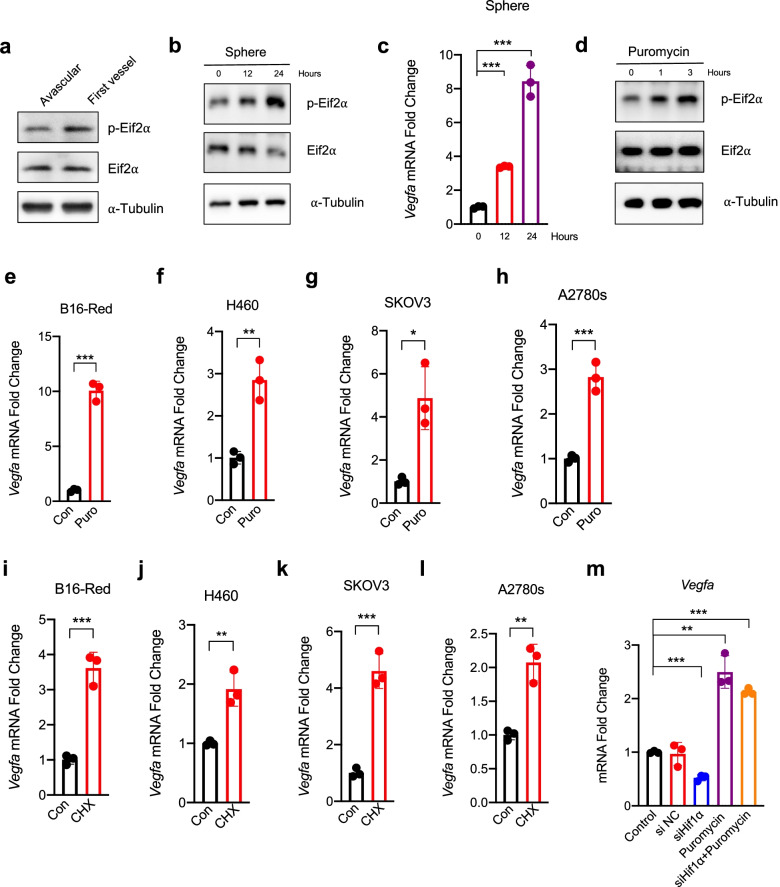
The reduction of protein translation promotes *Vegfa* expression independent of *Hif1α*. **a** Western blot analysis of the p-Eif2α, Eif2α, and α-tubulin of B16-Red transplant microtumor at avascular (about 12 h)and first vessel stage (about 24 h) (*n* = 50). **b** Western blot analysis of the p-Eif2α, Eif2α, and α-tubulin of B16-Red tumor spheroids at 0, 12 and 24 h. **c** RT-qPCR quantification of *Vegfa* expression in B16-Red tumor spheroids at 0, 12 and 24 h. **d** Western blot analysis of the p-Eif2α, Eif2α, and α-tubulin of puromycin (2 μg/mL) treated B16-Red cells at 0, 1 and 3 h. **e–h** RT-qPCR quantification of *Vegfa* expression in puromycin (4 μg/mL) treated B16-Red cells or in puromycin (1 μg/mL) treated H460, SKOV3 and A2780s cells for 24 h. **i-l** RT-qPCR quantification of *Vegfa* expression in cycloheximide (800 μM) treated B16-Red and H460 or in cycloheximide (200 μM) treated SKOV3 and A2780s cells for 24 h. **m** RT-qPCR quantification of *Vegfa* expression in B16-Red cells. Untreated (Control), negative control or *Hif1α* siRNA transfected for 48 h (siNC or siHif1α), puromycin (2 μg/mL) treated for 24 h (Puromycin), and puromycin (2 μg/mL) treated for 24 h and *Hif1α* siRNA transfected for 48 h (siHif1α + Puromycin). Control, Con. Puromycin, Puro. Cycloheximide, CHX. Unpaired t-test, *P < 0.05, ** *P* < 0.01, *** *P* < 0.001 (*n* = 3)

p-Eif2α activity is a critical inhibitor of protein synthesis and indirectly increases VEGFA expression [32–34]. Western blotting showed that the p-Eif2α was increased in microtumors during the angiogenic switch (Fig. 6a), while the expression of *Vegfa* was increased significantly (Fig. 4 a-b). We cultured B16-Red tumor spheroids to mimic the initial growth of microtumors and treated B16-Red cells with puromycin to inhibit protein translation. In B16-Red tumor spheroids, p-Eif2α was increased with the prolongation of culture time (Fig. 6b), indicating that protein translation was inhibited in tumor spheroids. We also examined expression of *Vegfa* in tumor spheroids and a significant increase (Fig. 6c). In puromycin-treated B16-Red cells, p-Eif2α was increased with prolongation of the treatment time (Fig. 6d). Expression of *Vegfa* in B16-Red, H460, SKOV3, and A2780 cell lines was also significantly increased after inhibition of protein translation by puromycin (Fig. 6e-h). Additionally, expression of *Vegfa* in B16-Red, H460, SKOV3 and A2780 cells was significantly increased after inhibition of protein translation by cycloheximide (Fig. 6i-l). These results suggest that the reduction of protein translation in microtumors during the angiogenic switch, tumor spheroids, and puromycin-treated tumor cells promoted *Vegfa* expression. Furthermore, knockdown of *Hif1α* did not affect the increase of *Vegfa* transcription induced by puromycin-mediated inhibition of protein translation (Fig. 6m). Thus, the reduction of protein translation promoted *Vegfa* expression, which did not depend on *Hif-1α*.

### Inhibition of protein translation by puromycin promotes the angiogenic switch depending on *Vegfa*

**Fig. 7:**
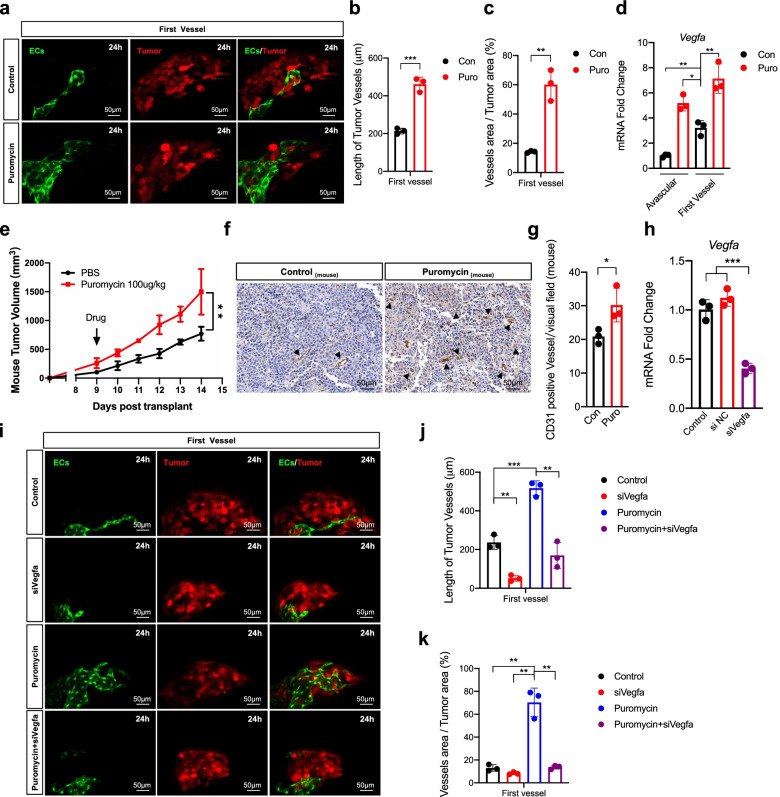
Inhibition of protein translation by puromycin promotes angiogenic switch depending on *Vegfa*. **a** Imaging of the first vessel sprouting of B16-Red transplant microtumors treated with puromycin (2 μg/mL) for 24 h before transplant. Scale bar, 50 μm. **b** and **c** The length of vessels and the ratio of vessels area versus tumor area of the first vessel. **d** RT-qPCR quantification of the *Vegfa* expression in the puromycin treated microtumors during the angiogenic switch(about 12 h and 24 h). **e** The mouse tumor volume of puromycin (100ug/Kg, intratumor) treated tumors. **f** CD31 staining of mouse tumors on day 14. Black arrow, CD31 positive tumor vessels. Scale bar: 50 μm. **g** The density of CD31 positive mouse tumor vessels per visual field. **h** RT-qPCR quantification of the *Vegfa* expression in B16-Red cells transfected with *Vegfa* siRNA (100 μM) for 48 h (*n* = 3). NC, negative control. **i** Imaging of the first vessel sprouting of B16-Red transplant microtumors treated with puromycin (2 μg/mL) for 24 h, transfected with *Vegfa* siRNA (100 μM) for 48 h, and treated with puromycin (2 μg/mL) for 24 h and transfected with *Vegfa* siRNA (100 μM) for 48 h before transplant. Scale bar, 50 μm. **j** and **k** The length of vessels and the ratio of vessels area versus tumor area of B16-Red transplant microtumor of the first vessel. ECs are green. Tumor is red. Control, Con. Puromycin, Puro. Unpaired t-test, *P < 0.05, ** *P* < 0.01, *** *P* < 0.001 (*n* = 3)

To determine the function of inhibiting protein translation in promoting the tumor angiogenic switch in vivo, we treated B16-Red cells with puromycin to inhibit protein translation and established the zebrafish angiogenic switch model with these cells (Fig. 7a). Both the length of tumor vessels and the ratio of the vessel area versus the tumor area were increased (Fig. 7b-c). Additionally, expression of *Vegfa* was significantly increased in puromycin-treated microtumors during the angiogenic switch (Fig. 7d).In the mouse xenograft model, tumors treated with puromycin grew faster (Fig. 7e) and showed a significantly higher platelet endothelial cell adhesion molecule (CD31)-positive vessel density than those in the control (CD31 was constitutively present on endothelial linings in vivo) (Fig. 7f-g). We then knocked down *Vegfa* in B16-Red cells with siRNA (Fig. 7h, Supplementary Fig. 7) and found that *Vegfa* knockdown inhibited the angiogenic switch, whereas puromycin promoted it (Fig. 7i). However, *Vegfa* knockdown while simultaneously treating tumor cells with puromycin eliminated the effect of promoting the angiogenic switch (Fig. 7i). The length of tumor vessels and the ratio of the vessel area versus the tumor area were increased when tumor cells were treated with puromycin, but *Vegfa* knockdown eliminated these increases (Fig. 7j-k). Additionally, we knocked out *Eif2α* (Supplementary Fig. 8a) and established the zebrafish angiogenic switch model with these cells (Supplementary Fig. 8b). We found that both the length of tumor vessels and the ratio of the vessel area versus the tumor area were increased (Supplementary Fig. 8c-d). Moreover, *Vegfa* expression was significantly increased during the angiogenic switch (Supplementary Fig. 8e). Therefore, inhibition of protein translation promoted the angiogenic switch depending on *Vegfa*.

## Discussion

Tumor neovascularization is a highly complex process including multiple steps. Understanding this process, especially the initial stage, has been limited by the difficulties of real-time visualizing the neovascularization of tumor tissues in living animal models. In our study, we have established a xenograft model in zebrafish by implanting mammalian tumor cells into the perivitelline space of Tg(*flk1:EGFP*) zebrafish embryos. This model provides a unique window for us to clearly visualize the process of the tumor angiogenic switch, the first new vessel sprouting from the host vessels into tumor mass (the transition from the avascular to the vascular stage), without surgical or other invasive procedures.

Hypoxia and HIF signaling are believed to be the major trigger of the angiogenic switch. However, all experimental evidence is indirect and there is no direct, real-time and dynamic in vivo evidence about if the hypoxia or HIF is necessary for the angiogenic switch, mainly due to the lack of suitable experimental models. The major mechanisms of tumor angiogenesis are conserved between fish and mammals. Systemic hypoxia could enhance the expression of angiogenic factor VEGFA to a high level when zebrafish embryos were exposed to a 7.5% oxygen level [35]. Interestingly, in our study, using our zebrafish angiogenic switch model, we found that hypoxia or HIF is not necessary for the angiogenic switch. This is consistent with previous studies that the HIF-deficient mouse embryos showed defects in blood vessel formation, but there is still some blood vessel formation in the embryos [36–38].

VEGF is a hypoxia-regulated gene via binding of HIF to its promoter under physiological and pathological conditions [10, 29]. In this study, we also found that *Vegfa* plays a crucial role in the angiogenic switch. However, the hypoxia or HIF did not work. Nowadays, more and more research suggests that VEGF expression can be induced by HIF independent pathway. Transcriptional up-regulation of VEGF by the unfolded protein response pathway involves activation of transcription factors, spliced x-box binding protein 1, activating transcription factor 4 (ATF4) and cleaved ATF6 respectively independent of HIF1α [39]. Amino acid restriction promotes VEGF expression, and capillary density in vivo via the general control nonderepressible 2/ATF4 independent of hypoxia or HIF1α [34]. VEGF expression can also be induced by the transcriptional co-activator peroxisome proliferator-activated receptor γ coactivator 1α through an oestrogen related receptor α dependent, HIF1α independent pathway [40]. Thus, there should be a HIF independent regulation of the expression of VEGF during the angiogenic switch.

The global rate of cellular protein synthesis is regulated by various signals and can contribute to angiogenesis. Dietary restriction correlates with diminished global translation [41]. Amino acid restriction promoted VEGF expression and resulted in increased vascular density in skeletal muscle [34]. This suggests that decreased protein translation may be related to angiogenesis. The aminoacyl-tRNA synthetases are exquisitely adapted to covalently link a single standard amino acid to its cognate set of tRNA isoacceptors and function in the first step of protein translation [30]. Aminoacyl-tRNA synthetase deficiency promotes angiogenesis via the unfolded protein response pathway dependent up-regulation of *Vegfa* [42]*.* The eIF2α has tightly controlled the initiation of protein synthesis. Phosphorylation of eIF2α enhanced its affinity for eIF2B, which sequestered p-eIF2α into an inactive complex, disrupted ternary complex formation [43–45], and inhibited global translation initiation [46]. p-eIF2α repress the translation of most mRNAs but selectively increase the translation of ATF4 [33], which can up-regulation the expression of VEGFA [34, 39]. And previous studies have found that p-eIF2α in 3D tumor spheroids increased compared to monolayer cells [47], which suggested that tumor cells cultured into spheroids resulted in a decrease in protein translation. Therefore, inhibition of protein translation may promote angiogenic switch because it can promote VEGFA expression. However, there is no direct evidence that whether the inhibition of protein translation affects the angiogenic switch. In our study, we showed it directly for the first time with the zebrafish angiogenic switch model that inhibition of cellular translation promotes the angiogenic switch by increasing *Vegfa* transcription. It is worth noting that data of our study are mainly presented from one cell line, so the results may have some limitations. But, we think this is an interesting and important supplement to the mechanism of the angiogenic switch. It is interesting to uncover more molecular details about how inhibited protein translation promotes *Vegfa* transcription and the angiogenic switch.

## Materials and methods

### Zebrafish husbandry

Tg(*flk1: EGFP*) transgenic zebrafish were maintained in a normal condition of 28 °C, pH 7.2∼7.4, 14 h on and 10 h off light cycle [48, 49].

### Cell and cell culture

The mouse skin melanoma line B16, the human non-small cell carcinoma cell line H460, and the human ovarian cancer cell line A2780s and SKOV3 were obtained from American Type Culture Collection and cultured at 37 °C in 5% CO_2_ in DMEM or RPMI-1640 supplemented with 10% fetal bovine serum. The red fluorescence-labelled B16 cells (B16-Red) were generated with pCMV-DsRed-express (Clontech, USA) [26].

### Mouse and tumor inoculation

C57BL/6 female mice are purchased from the laboratory animal centre (Sichuan University, China). At 5 to 6 weeks of age, mice were subcutaneously injected with 5 × 10^5^ B16-Red cells. About 9 days after inoculation, the intratumor injection of phosphate buffer saline or puromycin (100 μg/Kg) was started and lasted for 5 days. Then the mice were sacrificed and transplant tumors were collected for staining. All animal work has been approved by Sichuan Animal Care and Use Committee and conducted based on relevant guidelines. The Permit Number is SYXK (Chuan) 2008–119.

### Establishment of zebrafish angiogenic switch model

Cells were harvested at a concentration of 1 × 10^7^ cells/ml. This mixture was loaded into a borosilicate glass needle. 5∼10 nanoliters suspension were implanted into the perivitelline space of each zebrafish embryo (about 48 h) and 15∼20 fish were selected for each group. Then, based on visual observations, microtumors in two states were selected to simulate the process of the ‘angiogenic switch’.

### Imaging

Living zebrafish embryos were anaesthetized by 0.003% tricaine and embedded in a sagittal plane in a 1.5% low melting point agarose. Digital micrographs were taken with a Zeiss 880 Confocal microscope or a Leica TCS SP8 confocal microscope.

### Recording of the survival of zebrafish angiogenic switch model

After the establishment of the zebrafish angiogenic switch model, 20 fish were selected for each group and recorded the survival situation daily. The zebrafish were maintained in a normal condition of 28 °C and the observation lasted for 10 days. The zebrafish fed with the ground brine shrimp from 4 days post-injection (6 days post-fertilization).

### Quantitative analysis of neovascularization in tumor xenografts

Measurement was done on the zebrafish digital micrographs. The vessel length and area were quantified by ImageJ software. The number of vessel branches and CD31 positive vessels was quantified by manual counting.

### Transcriptome sequencing and analysis

The microtumor samples in two states prepared by microsurgery were sent to Annoroad Gene Technology (Beijing, China) for transcriptome sequencing. In one state, the tumor has not yet induced angiogenesis, which means ‘angiogenic switch’ is off (about 12 h); in another, the tumor induces the sprouting of the first vessel, which means ‘angiogenic switch’ is on (about 24 h). After the construction of a single cell transcriptome library, libraries were sequenced on Illumina HiSeq 2500 V4 and HiSeq 4000 platform with PE125 and PE150. The sequencing data were analysed by the R package. The gene set enrichment analysis (GSEA) was down by GSEA Software.

### Plasmid vector construction

Single guide RNAs targeting murine *Vegfa* and *Eif2*α (Supplementary Table 1) [50] were cloned into LentiCRISPRv2 plasmid (AddGene, USA) with BsmbI restriction site.

#### Plasmid vector and small interfering RNA transfection

The *Vegfa* expression (pcDNA3.1-VEGFA) and knockout vector were transfected with Lipofectamine 3000 reagent (Invitrogen, USA) for 48 h, followed by Puromycin selection (4 µg/mL) for 48 h. The small interfering RNA (siRNA) for *Vegfa and Hif1α* (RiboBio, China) were also transfected with Lipofectamine 3000 reagent for 48 h. The siRNA targets were used (Supplementary Table 2).

#### Detection of tumor hypoxia and target genes expression

B16 cells were cultured for about 24 h. Then cells were incubated with Image-iT™ Red Hypoxia Reagent (Invitrogen, USA) at a final concentration of 5 µM for 1 h and implanted into the perivitelline space of the zebrafish embryo. At the same time, part of the incubated cells was kept and cultured under normoxic or hypoxic (1%). The fluorescent signal of hypoxia was detected by a confocal microscope. The microtumor samples were put into cell lysis buffer (Signosis, USA) immediately after microsurgery. And the reverse transcription and real-time quantitative polymerase chain reaction (qPCR) were performed according to the instructions to detect target genes expression.

#### Hematoxylin–eosin staining and immunohistochemical staining

Euthanized fish were fixed with 4% paraformaldehyde and followed by dehydration in gradient ethanol and xylene. Then fish were embedded in paraffin and sectioned (5 μm) sagittally [51]. Sections were deparaffinized and rehydrated through graded ethanol, which was then stained with hematoxylin–eosin (H&E). CD31 immunohistochemical staining was performed by Servicebio Technology (Wuhan, China).

#### 3D tumor spheroids culture

Tumor spheroids were created with the 0.5% methylcellulose-culture medium containing 1 × 10^6^ cell/mL. The tumor cell suspension was pipetted into the non-treated 6-cell well dishes and incubated for 4∼6 h. Then, the resultant cell aggregates were cultured at 37 °C in 5% CO_2_.

#### Pharmacological treatment of tumor cells with puromycin or cycloheximide

Puromycin and cycloheximide were added directly into the culture media at a final concentration of 2 µg/mL puromycin for B16-red, 1 µg/mL puromycin for H460, A2780 and SKOV3, 800 µM cycloheximide for B16-Red and H460 or 200 µM cycloheximide for A2780 and SKOV3 to inhibit the translation. Pharmacological treatment of tumor cells was maintained for 24 h.

#### Gene expression detection with quantitative reverse transcription-polymerase chain reaction (RT-qPCR)

Total RNA of tumor cells or spheroids was isolated by the Trizol reagent (Invitrogen, USA) and cDNA synthesis was performed by the PrineScript^TM^RT reagent Kit (Takara, Japan) according to the instructions. qPCR was performed with SYBR labelled qPCR mix (Bio-Rad, USA). Expression values were normalized to *actb* expression. All qPCR primers were used (Supplementary Table 3).

#### Western-blot

B16-red cells, spheroids or microtumors were lysed with RIPA lysate. Total proteins were separated on SDS–polyacrylamide gels and transferred to a PVDF membrane. The protein of interest was identified by incubating with the target antibody. α-Tubulin (Beyotime Biotechnology, China) was detected as the internal control of Eif2α, phosphorylated Eif2α (p-Eif2α) (Huabio, China), Vegfa (Servicebio, China).

#### Statistical analysis

Statistical analysis was done by Prism software with unpaired student’s t-test, α = 0.05, 0.01 or 0.001. It’s regarded as statistically significant (*P* < 0.05).

## Supplementary information

**Additional file 1.**

## Data Availability

The datasets generated and/or analyzed during the current study are available from the corresponding author upon reasonable request.

## Abbreviations

HIF: Hypoxia-inducible factor
VEGFA: Vascular endothelial growth factor A
FGF: Fibroblast growth factor
PDGF: Platelet-derived growth factor
EGFP: Enhanced green fluorescent protein
dpf: Days post fertilization; dpi: days post-injection
p-Eif2α: Phosphorylated eukaryotic translation initiation factor 2 alpha
PECAM-1/CD31: Platelet endothelial cell adhesion molecule
ATF4: Activating transcription factor 4
GSEA: Gene set enrichment analysis
GO: Gene ontology; sgRNA: single guide RNAs
siRNA: Small interfering RNA
H&E: Hematoxylin–eosin
B16-Red: Red fluorescence-labelled B16 cells
TC: Ternary complex
