# Supplementary material for: The inhibition of protein translation promotes tumor angiogenic switch
Source: Mol Biomed. 2022 Jun 13;3:18. doi: 10.1186/s43556-022-00081-4 (PMC9192909; doi:10.1186/s43556-022-00081-4)
Supplement: Supplementary file 1 — Additional file 1. [file 43556_2022_81_MOESM1_ESM.pdf]

Supplemental Information

The Inhibition of Protein Translation but not HIF Promotes Angiogenic Switch in Zebrafish Mammalian Xenograft

Hui Luo[1,#], Yuge Shen[1,#], Weiting Liao[1,#], Qiqi Li[1], Ni Wu[2], Jian Zhong[1], Chaoxin Xiao[1], Jia Gan[1], Yun Yang[1], E Dong[1], Guimin Zhang[1], Binrui Liu[1], Xiaozhu Yue[1], Lin Xu[1], Yan Liu[1], Chengjian Zhao[1], Qian Zhong[3,*], Hanshuo Yang[1,4,*]

[1] State Key Laboratory of Biotherapy and Cancer center, West China Hospital, Sichuan University and Collaborative Innovation Center, Chengdu, China.
[2] The Third Affiliated Hospital of Chengdu Medical College, Pidu District People's Hospital, Chengdu, China.
[3] Department of Gynecology and Obstetrics, West China Second University Hospital of Sichuan University, Chengdu, China.
[4] Experimental and Research Animal Institute, Sichuan University, Chengdu, China.
[*] Corresponding authors. Department of Gynecology and Obstetrics, West China Second University Hospital of Sichuan University, Chengdu, China. Email: cdcd91761@163.com. State Key Laboratory of Biotherapy and Cancer center, West China Hospital, Sichuan University, No.17 Renmin South Road Section Three, Chengdu, Sichuan, 610041, China. Email: yhansh@scu.edu.cn.
[#] These authors contributed equally to this article.

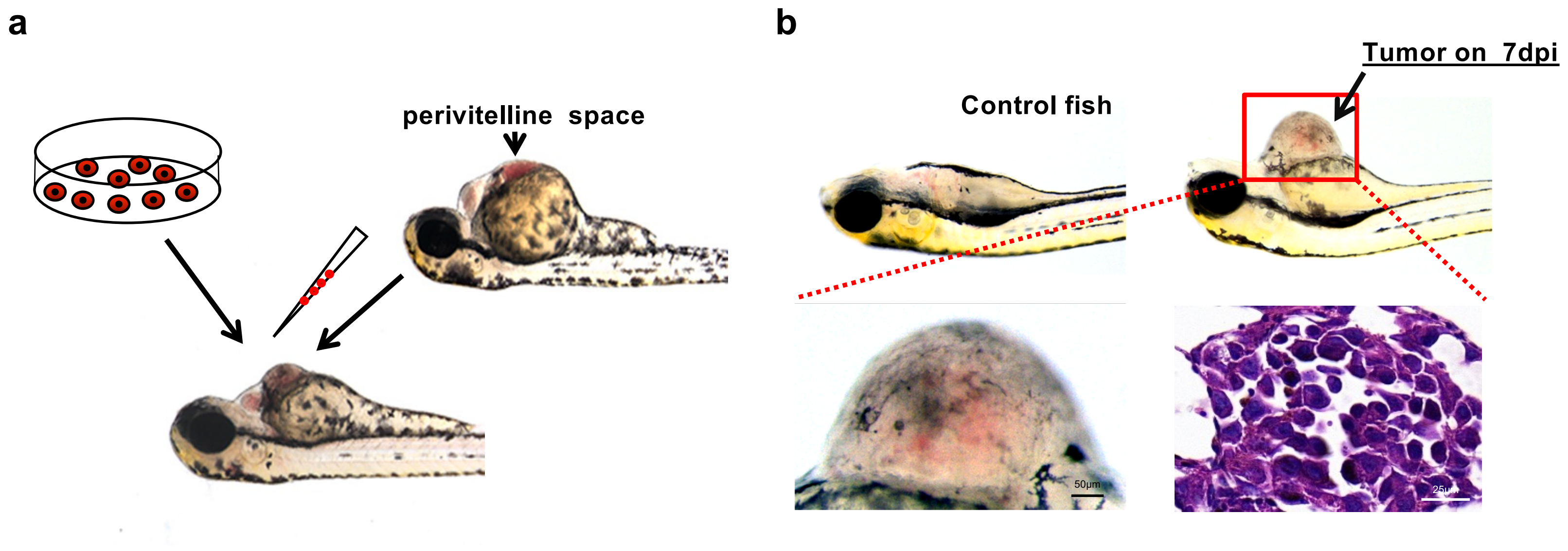

Fig S1. Mammal xenograft model in zebrafish. a. Schematics indicating inoculate tumor cells into the perivitelline space of zebrafish larvae. **b.** H&E staining of zebrafish tumor model after 7 dpi. Scale bar: black 50μm, white 25μm

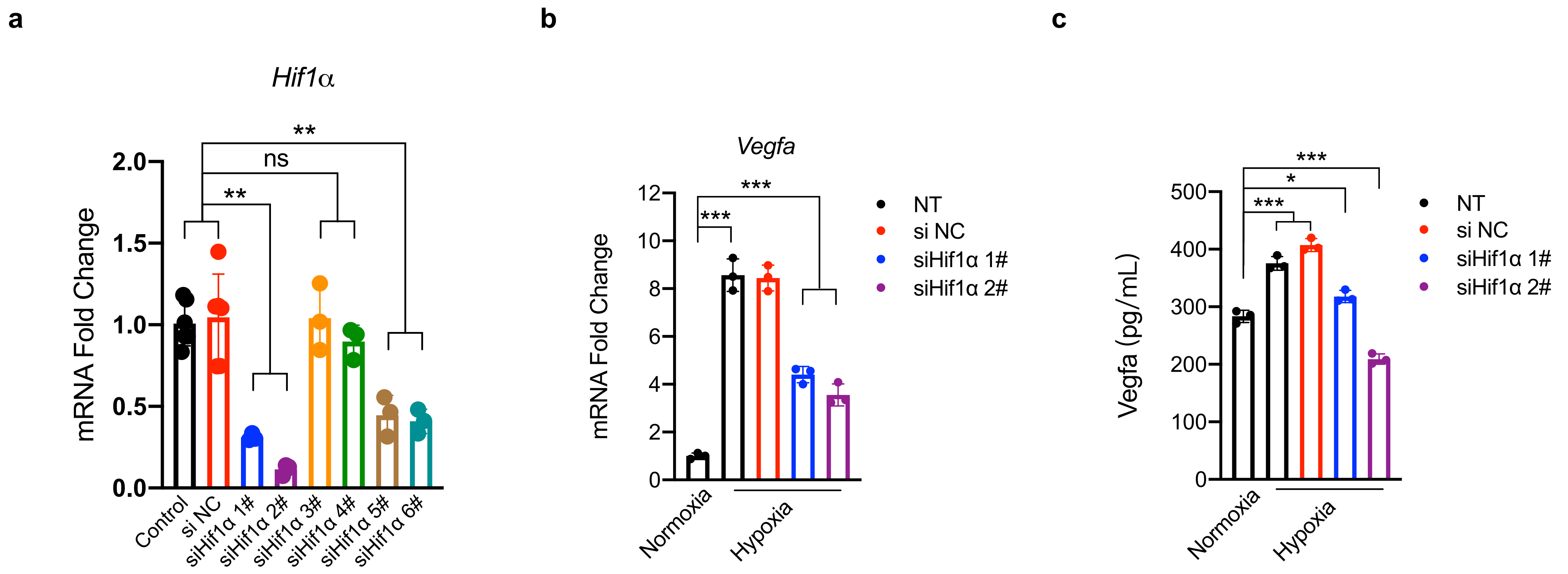

Fig. S2 Knockdown the *Hif1α* and its' effect on *Vegfa* expression and protein levels under hypoxia. a. RT-qPCR quantification of the *Hif1α* expression in B16-Red cells transfected with Hif1α siRNA or negative control siRNA for 48 hours (n=3 or 6). NT, non-transfected. NC, negative control. **b**. RT-qPCR quantification of the *Vegfa* expression in B16-Red cells transfected with Hif1α siRNA (50μM) under normoxia or hypoxia (1%) for 48 hours (n=3). **c**. Enzyme linked immunosorbent assay (Elisa) quantification the protein levels of *Vegfa* in B16-Red cells transfected with Hif1α siRNA (50μM) under normoxia or hypoxia (1%) for 48 hours (n=3). Unpaired t test, ns *P*>0.05, **P*<0.05, ** *P*<0.01, *** *P*<0.001.

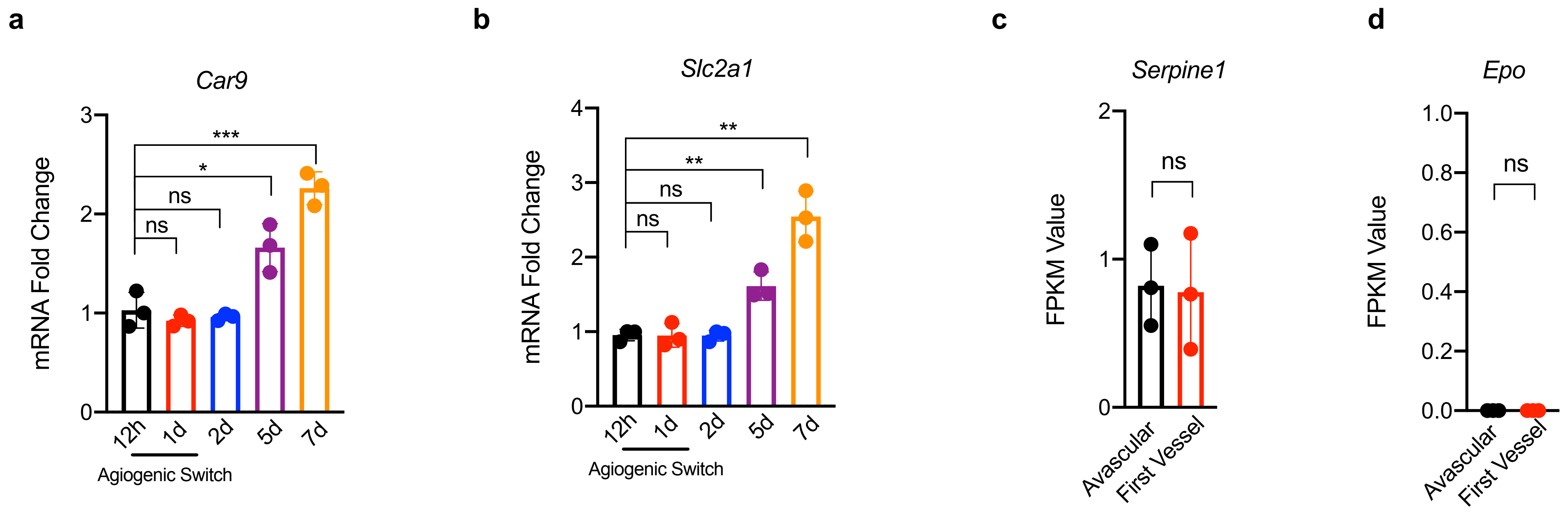

Fig. S3 The expression of Hif1α target genes during the angiogenesis. a. and **b**. RT-qPCR quantification of the Car9/CA9 and Slc2a1/GLUT1 expression in the transplant microtumors during the angiogenesis(n=3). **c**. and **d**. The change of fragments per kilobase of exon model per million (FPKM) of RNAseq data for HIF2 target genes *Serpine1* and *Epo* during the angiogenic switch. Unpaired t test, ns $P>0.05$, *$P<0.05$, ** $P<0.01$, *** $P<0.001$..

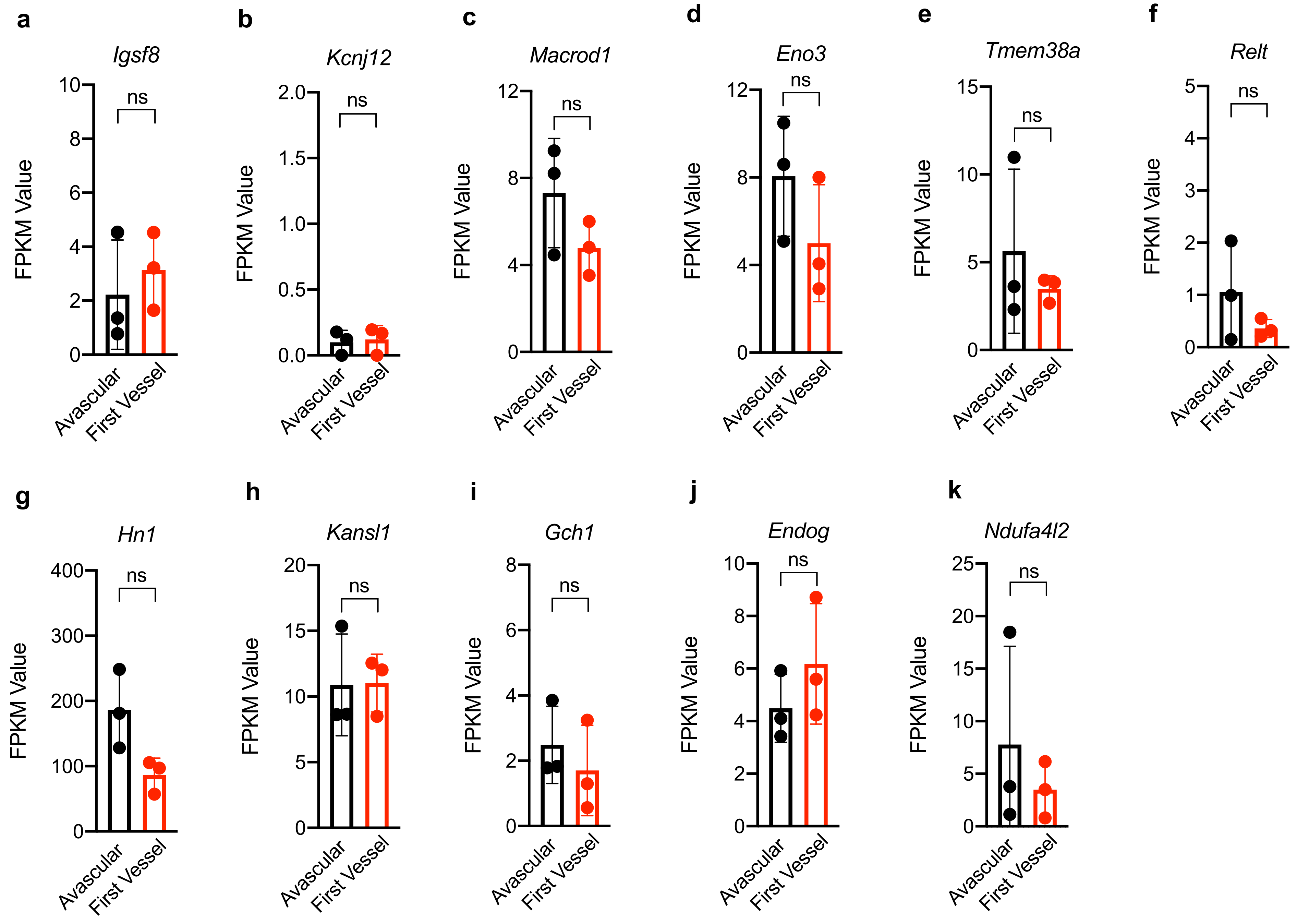

Fig. S4 The expression of Hif target genes during the angiogenic switch. a-k. The change of fragments per kilobase of exon model per million (FPKM) of RNAseq data for HIF target genes during the angiogenic switch (n=3). Unpaired t test, ns *P*>0.05.

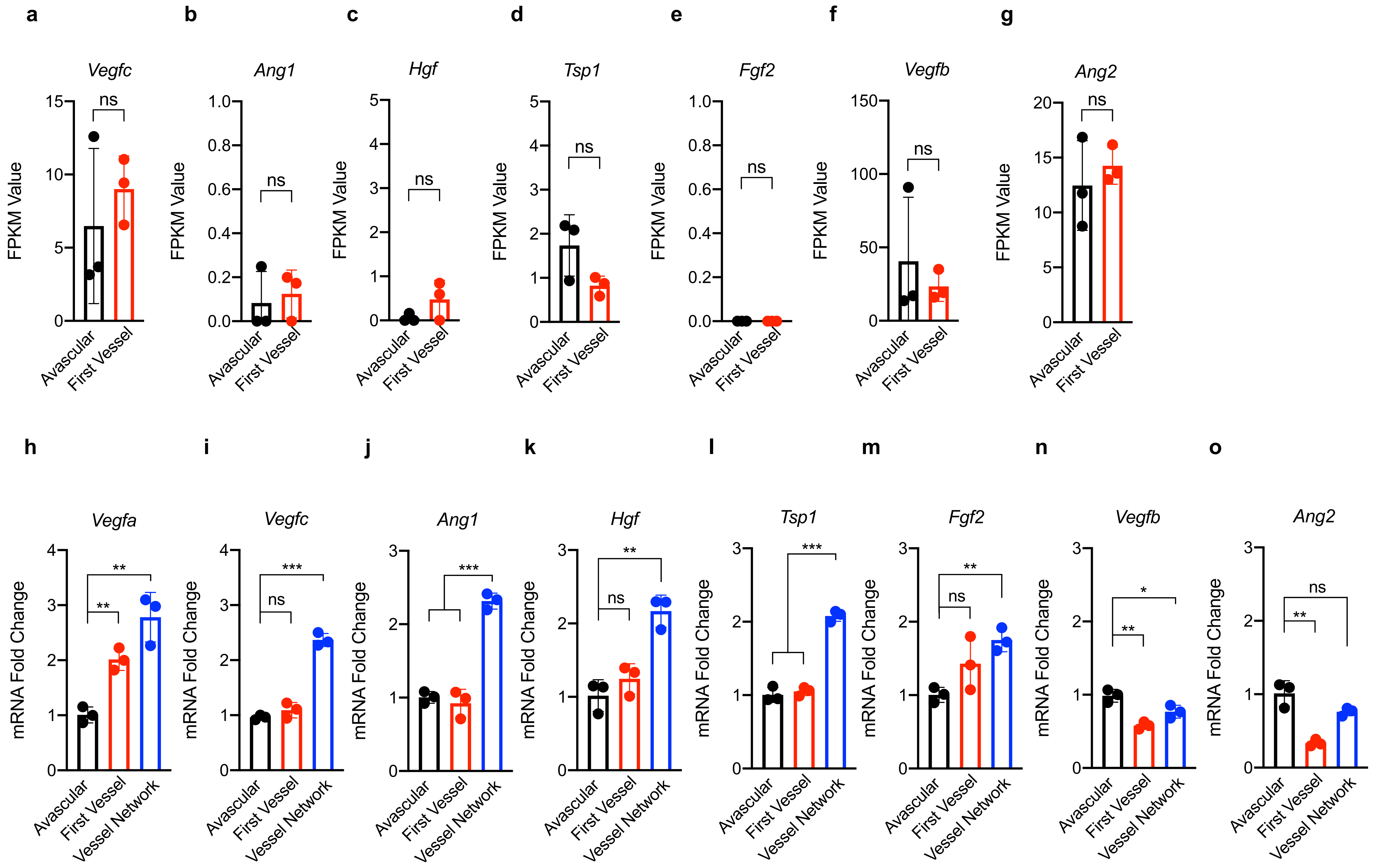

Fig. S5 The expression of angiogenesis related genes in different stage. a-g. The change of fragments per kilobase of exon model per million (FPKM) of angiogenesis related genes during the angiogenic switch (n=3) (about 12 hours and 24 hours). **h-p**. RT-qPCR quantification of the angiogenesis related genes expression during the angiogenesis (n=3) (about 12 hours, 24 hours and 72 hours). Unpaired t test, ns *P*>0.05, **P*<0.05, ** *P*<0.01, *** *P*<0.001.

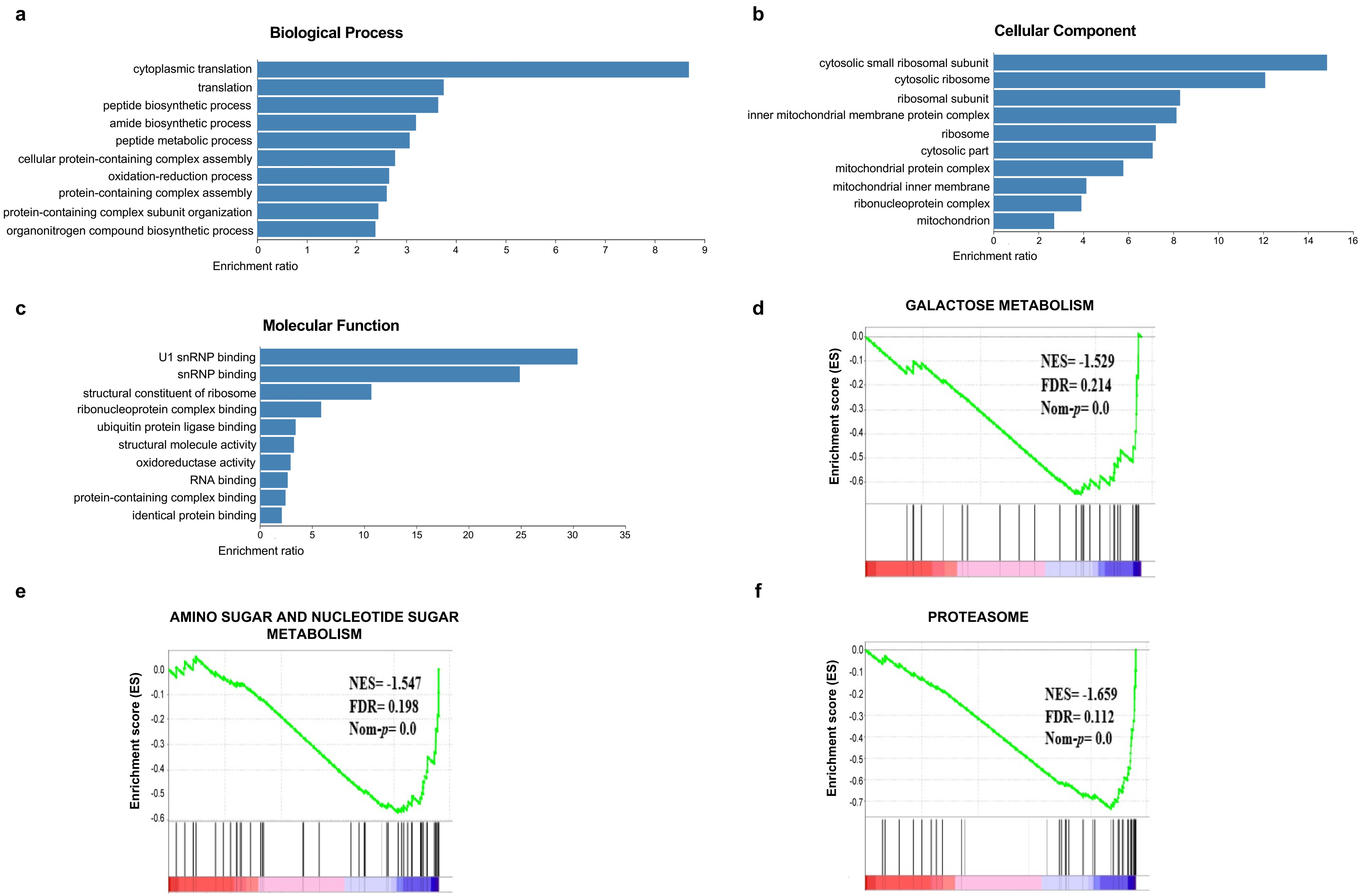

Fig. S6 The protein translation of microtumors decreases during the angiogenic switch. a-c. The GO analysis of transcriptome during the angiogenesis switch (n=3). **d-e**. The GSEA analysis of transcriptome for the galactose metabolism pathway (NES-1.529, FDR 0.214, Nominal p-value 0.0), amino sugar and nucleotide sugar metabolism pathway (NES-1.547, FDR 0.198, Nominal p-value 0.0) and proteasome pathway (NES-1.659, FDR 0.112, Nominal p-value 0.0).

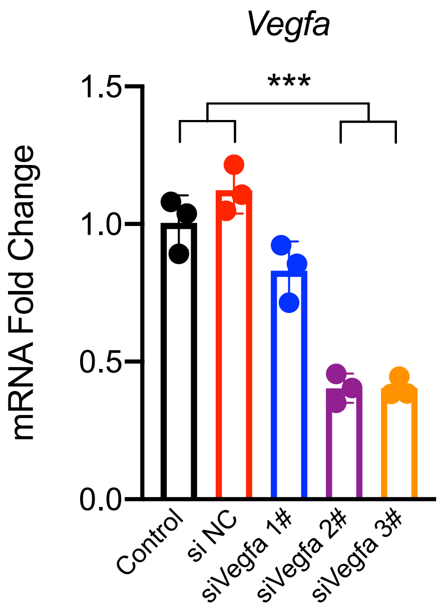

Fig. S7 Knockdown the *Vegfa* in B16-Red cells by small interfering RNA. RT-qPCR quantification of *Vegfa* expression in B16-Red tumor cells transfected with Vegfa siRNA 1#, 2#, 3# or negative control siRNA for 48hours (n=3). NC, negative control. Unpaired t test, ****P*<0.001.

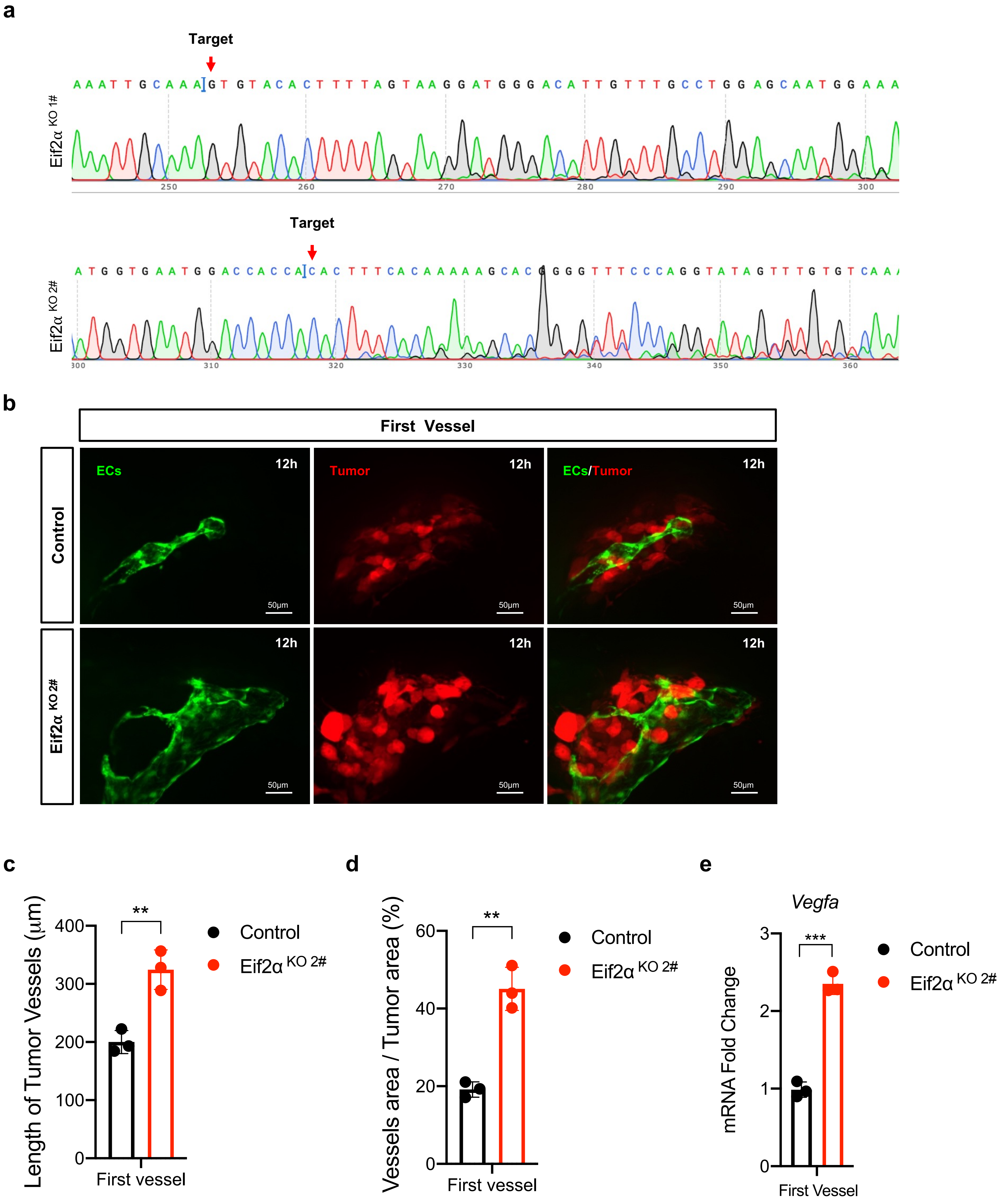

Fig. S8 Inhibition of protein translation by knockout *Eif2α* promotes angiogenic switch and increases the expression of *Vegfa*. a. Sequencing results of DNA sequence near *Eif2α* knockout targets after *Eif2α* knockout in B16-Red tumor cells. **b**. Imaging of the first vessel sprouting of B16-Red transplant microtumors knocked out *Eif2α* before transplant with confocal microscope. Scale bar, 50μm. **c**. and **d**.The length of vessels and the ratio of vessels area versus tumor area of the first vessel(n=3). **e**. RT-qPCR quantification of *Vegfa* expression in B16-Red microtumors knocked out *Eif2α* in the first vessel stage(n=3). Unpaired t test, ** P<0.01, *** P<0.001.

Supplementary Table 1 The single guide RNA targets for *Vegfa* and *Eif2α*.

Single guide RNA targets	Sequence(5'-3')
sgVegfa 1#	TCGGACGGCAGTAGCTTCGC
sgVegfa 2#	CCGTCCGATTGAGACCCTGG
sgEif2α 1#	GTGTACACTTTTAGTAAGGA
sgEif2α 2#	ACCGTGCTTTCTGTGAAGTG

Supplementary Table 2 The siRNA targets for *Vegfa* and *Hif1α*.

siRNA targets	Sequence(5'-3')
siVegfa 1#	GCACATTGGCTCACTTCCA
siVegfa 2#	GGAAGAAGAGGCCTGGTAA
siVegfa 3#	GGCTTACCCTTCCTCATCT
siHif1α 1#	GGTATGTGGCATTTATTTG
siHif1α 2#	CCAGUUACGAUUGUGAAGUUATT
siHif1α 3#	CAAGCAACTGTCATATATA
siHif1α 4#	CTGATAACGTGAACAAATA
siHif1α 5#	CCCAUUCCUCAUCCGUCAAAUTT
siHif1α 6#	CCCAGUGAAUAUUGCUUUGAUTT
siNC	UUCUCCGAACGUGUCACGUTT

Supplementary Table 3 The primers for RT-qPCR.

Primer	Sequence(5'-3')
Vegfa-Forwad	AGGGCAGAATCATCACGAAGT
Vegfa-Reverse	AGGGTCTCGATTGGATGGCA
Vegfb-Forwad	GCCAGACAGGGTTGCCATAC
Vegfb-Reverse	GGAGTGGGATGGATGATGTCAG
Vegfc-Forwad	GAGGTCAAGGCTTTTGAAGGC
Vegfc-Reverse	CTGTCCTGGTATTGAGGGTGG
Fgf2-Forwad	GCGACCCACACGTCAAACTA
Fgf2-Reverse	TCCCTTGATAGACACAACTCCTC
Hgf-Forwad	ATGTGGGGGACCAAACTTCTG
Hgf-Reverse	GGATGGCGACATGAAGCAG
Angpt1-Forwad	CACATAGGGTGCAGCAACCA
Angpt1-Reverse	CGTCGTGTTCTGGAAGAATGA
Angpt2-Forwad	CCTCGACTACGACGACTCAGT
Angpt2-Reverse	TCTGCACCACATTCTGTTGGA
Thbs1-Forwad	GGGGAGATAACGGTGTGTTTG
Thbs1-Reverse	CGGGGATCAGGTTGGCATT
Hif1α-Forwad	TGACTGTGCACCTACTATGTCACTT
Hif1α-Reverse	GGTCAGCTGTGGTAATCCACTC
Car9(CA9)-Forwad	CTGAAGACAGGATGGAGAAG
Car9(CA9)-Reverse	GCAGAGTGCGGCAGAATG
Slc2a1(GLUT-1)-Forwad	GGGAGACGCATAGTTACAGC
Slc2a1(GLUT-1)-Reverse	CTCCCACAGCCAACATGAG
Actin-Forwad	GTTCGCCATGGATGACGATAT
Actin-Reverse	CTGGTGCCTAGGGCGGCCCA
Vegfa-Forwad (Human)	TAGGTGAGATGAGCTTCCTACA
Vegfa-Reverse (Human)	ATTTACACGTCTGCGGATCTT
Gapdh-Forwad (Human)	GAAGATCAAGATCATTGCTCCT
Gapdh-Reverse (Human)	TACTCCTGCTTGCTGATCCA
